# Complementary Role of BMI and EOSS in Predicting All-Cause and Cause-Specific Mortality in People with Overweight and Obesity

**DOI:** 10.3390/nu16203433

**Published:** 2024-10-10

**Authors:** Fabio Bioletto, Valentina Ponzo, Ilaria Goitre, Beatrice Stella, Farnaz Rahimi, Mirko Parasiliti-Caprino, Fabio Broglio, Ezio Ghigo, Simona Bo

**Affiliations:** 1 Endocrinology, Diabetes and Metabolism, Department of Medical Sciences, University of Torino, 10126 Torino, Italy; fabio.bioletto@unito.it (F.B.); fabio.broglio@unito.it (F.B.); ezio.ghigo@unito.it (E.G.); 2 Department of Medical Sciences, University of Torino, 10126 Torino, Italy; ilaria.goitre@unito.it (I.G.); simona.bo@unito.it (S.B.); 3 Dietetic and Clinical Nutrition Unit, Città della Salute e della Scienza Hospital, C.so Bramante 88, 10126 Torino, Italy; frahimi@cittadellasalute.to.it

**Keywords:** body mass index, EOSS, obesity, mortality

## Abstract

Objective: To assess the complementary role of the Body Mass Index (BMI) and Edmonton Obesity Staging System (EOSS) in predicting all-cause and cause-specific mortality in people living with overweight and obesity (PLwOW/O). Methods: A longitudinal analysis of prospectively collected data from the 1999–2018 cycles of the National Health and Nutrition Examination Survey (NHANES) was conducted. The association between BMI, EOSS, and mortality was evaluated through Cox regression models, adjusted for confounders. Results: The analysis included 36,529 subjects; 5329 deaths occurred over a median follow-up of 9.1 years (range: 0–20.8). An increased mortality risk was observed for obesity class II and III (HR = 1.21, 95% CI 1.08–1.36, *p* = 0.001 and HR = 1.58, 95% CI 1.39–1.80, *p* < 0.001; compared to overweight), and for EOSS stage 2 and 3 (HR = 1.36, 95% CI 1.16–1.58, *p* < 0.001 and HR = 2.66, 95% CI 2.26–3.14, *p* < 0.001; compared to stage 0/1). The prognostic role of BMI was more pronounced in younger patients, males, and non-Black individuals, while that of EOSS was stronger in women. Both BMI and EOSS independently predicted cardiovascular- and diabetes-related mortality. EOSS stage 3 was the only predictor of death from malignancy or renal causes. Conclusions: BMI and EOSS independently predict all-cause and cause-specific mortality in PLwOW/O. Their integrated use seems advisable to best define the obesity-related mortality risk.

## 1. Introduction

Obesity is a chronic, multifactorial, complex disease that impairs health, survival, and quality of life [1]. Its prevalence is continuously increasing worldwide [2], influenced by a variety of factors, such as changes in lifestyle habits and dietary patterns, which influence the risk of obesity and metabolic complications not only due to excessive caloric intake but also because of an increased consumption of foods with poor nutritional quality, such as ultra-processed foods [3,4].

The Body Mass Index (BMI) was widely used for classifying obesity due to its simplicity and cost-effectiveness [5], but its reliability has been increasingly questioned [6], as individuals with the same BMI may have a different body composition and health status [7]. The relationship of BMI with all-cause mortality remains controversial; several meta-analyses reported a positive association with BMI starting from values > 25 kg/m^2^ [8,9,10], while others showed that patients with overweight or class I obesity may face similar or even diminished risks for all-cause mortality compared to normal weight subjects [11,12,13]. While these findings could be influenced at least in part by the depletion-of-susceptibles bias, which might have skewed the results by prematurely removing the subjects at highest risk, they still highlight an unmet need for alternative tools to better stratify the risk of adverse outcomes in patients living with overweight and obesity (PLwOW/O).

The Edmonton Obesity Staging System (EOSS) is a 5-stage ordinal system that assesses the severity of obesity-related comorbidities to classify PLwOW/O into graded categories based on their health risk profile, without including BMI [14,15,16]. This staging approach has been reported to predict long-term weight loss [17] and the risk for polypharmacy and healthcare service use [18], as well as increased perioperative complications and mortality in patients undergoing bariatric surgery [18,19]. A few studies have investigated the ability of EOSS to predict mortality risk in PLwOW/O [20,21,22]. Out of 6224 patients living with obesity (PLwO) from the Aerobics Center Longitudinal Study, those in EOSS stage 2 or 3 showed a significantly higher risk of all-cause and cardiovascular-related mortality compared to normal-weight individuals, over a 16-year follow-up period [20]. An analysis of data from the 1988–1994 National Health and Nutrition Examination Survey (NHANES), which included 7967 PLwOW/O, demonstrated that EOSS was a strong predictor of all-cause mortality, independent of BMI [21]. To the best of our knowledge, however, no previous study has assessed the predictive role of EOSS on the risk of cause-specific mortality.

The aim of the present study was to evaluate the complementary role of BMI and EOSS in predicting all-cause and cause-specific mortality in a representative cohort of PLwOW/O extracted from the general U.S. population. The hypothesis was that both BMI and EOSS would independently predict mortality, supporting the utility of their integrated use in the prediction of adverse clinical outcomes in PLwOW/O.

## 2. Materials and Methods

### 2.1. Survey Design and Data Collection

A longitudinal analysis of prospectively collected data from the 1999–2018 cycles of the National Health and Nutrition Examination Survey (NHANES) was conducted [23], with the primary aim to determine the role of BMI and EOSS as predictors of all-cause and cause-specific mortality.

NHANES uses a stratified, multistage, clustered probability sampling design to generate a sample of participants representative of the general non-institutionalized U.S. population. Demographic information, socioeconomic status, and medical histories were collected through in-home interviews. Subsequently, study participants were invited to a mobile examination center (MEC) for standardized clinical assessments, which included anthropometric measurements, laboratory tests, and other instrumental examinations. A detailed description of the survey design and of the data collection methodology can be found on the NHANES website [23]. The original survey received approval from the Centers for Disease Control and Prevention Research Ethics Review Board “https://www.cdc.gov/nchs/nhanes/irba98.htm (accessed on 23 May 2024)” and was in accordance with the principles of the Declaration of Helsinki. Written informed consent was obtained from all adult participants.

### 2.2. Clinical Data and Laboratory Tests

Body measurements, including weight (kg), height (cm), and BMI (kg/m^2^), were obtained during the mobile examination center (MEC) visit. All anthropometric measurements were performed by trained health technicians, who completed a 2-day training program with survey staff and an expert anthropometrist. The training included an overview of the component, general guidelines and technical skills for each measurement, and demonstrations conducted by the expert examiner with volunteer subjects. During each survey cycle, scheduled equipment calibration was performed for all MECs by the health technicians and verified by supervisory staff. All measurements were recorded in strict accordance with the *Anthropometric Standardization Reference Manual* by Lohman et al. [24]. Complete details are reported in the Anthropometry Procedures Manuals available for each survey cycle on the NHANES website [23].

In the present analysis, in accordance with the World Health Organization (WHO) classification, BMI categories were defined as follows: underweight if BMI < 18.5 kg/m^2^; normal weight if BMI ≥ 18.5 kg/m^2^ and <25 kg/m^2^; overweight if BMI ≥ 25 kg/m^2^ and <30 kg/m^2^; class I obesity if BMI ≥ 30 kg/m^2^ and <35 kg/m^2^; class II obesity if BMI ≥ 35 kg/m^2^ and <40 kg/m^2^; and class III obesity if BMI ≥ 40 kg/m^2^. Information regarding pregnancy status, annual household income, education level, smoking status, and comorbidities was based on self-report using specific questionnaires. Laboratory methods for the measurement of all performed blood and urine tests are reported in detail on the NHANES website [23].

### 2.3. EOSS Stage Classification

An EOSS score was assigned to each adult with a BMI ≥ 25 kg/m^2^. The EOSS stage was assigned to participants using data extracted from the health interview, physical examination, and laboratory investigations, complying with the classification criteria originally proposed by Sharma et al. [11]. The exact operational criteria adopted for clinical and functional staging of each obesity-related comorbidity included in EOSS are outlined in the Supplementary Material (Table S1); the final EOSS score was determined based on the highest-ranked risk factor present for each individual. In case no data about a specific item were available, the patient was assumed not to be affected by that comorbidity. The final classification was stratified into 3 classes: stages 0/1 were considered as a single category and reflected no or subclinical weight-related medical conditions, respectively; stage 2 reflected the presence of established obesity-related chronic disease; and stage 3 reflected the presence of established end-organ damage. As already discussed in previous studies [21,22], the available NHANES data were insufficient to identify patients belonging to EOSS stage 4.

### 2.4. Mortality Ascertainment

Mortality data from death certificates from the National Death Index were linked to NHANES based on the participant sequence number available on both datasets. The present analysis is based on the public-use linked mortality files for the NHANES 1999–2018 cycles, which include follow-up time and cause of death for adult participants through 31 December 2019. Further details on the linkage method and analytic guidelines can be found on the specific webpage of the National Center for Health Statistics [25].

### 2.5. Sample Selection

**Figure 1:**
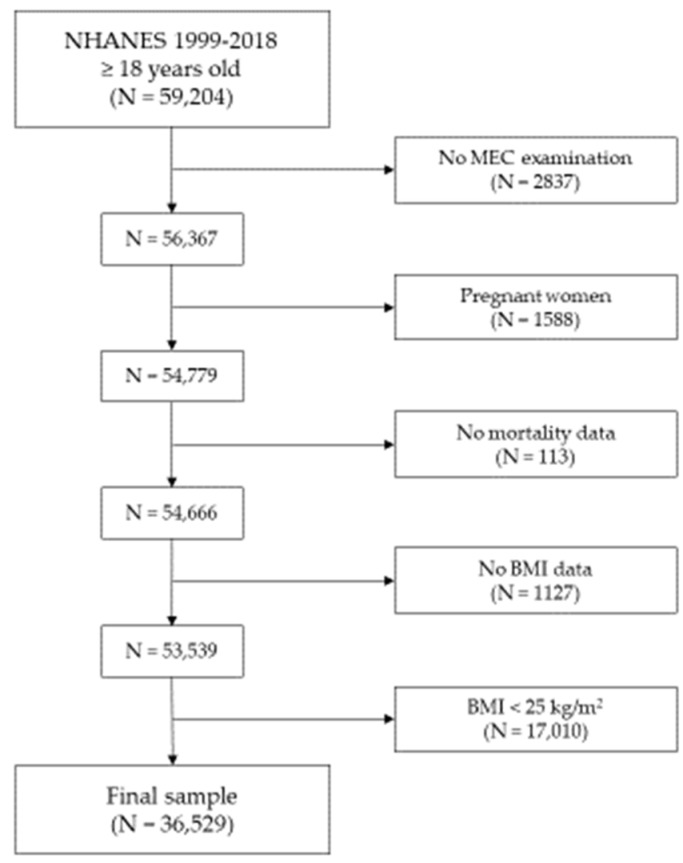
Flowchart of the participant selection from the NHANES 1999–2018 cohorts.

A total of 59,204 adult (≥18 years old) subjects participated in the 1999–2018 NHANES survey cycles. Among them, 2837 individuals in which an MEC visit was not performed and 1588 pregnant women were excluded. Furthermore, 113 participants with unavailable mortality data and 1127 with unavailable BMI data were excluded. Of the remaining 53,539 individuals, 17,010 were excluded because of a BMI < 25 kg/m^2^, leading to a final sample of 36,529 patients. The full process of sample selection is graphically summarized in Figure 1.

### 2.6. Statistical Analysis

All analyses were conducted using appropriate weighting, in order to account for the complex NHANES survey design. Data were summarized as weighted means and standard deviations (SDs) for continuous variables, and as weighted proportions for categorical ones. Patient characteristics according to BMI classes were compared using adjusted Wald tests for continuous variables and Rao–Scott chi-square tests for categorical variables.

The associations between BMI classes, EOSS stages, and all-cause mortality were assessed using multivariable Cox regression models adjusted for sex, age, ethnicity, income, educational level, and smoking status. The potential role of key demographic variables (sex, age, and ethnicity) as effect modifiers of the relationship between BMI classes, EOSS stages, and all-cause mortality was assessed by including interaction terms in the regression models and by testing the global significance of each interaction by the adjusted Wald test. Stratified analyses were then performed to further dissect the different prognostic roles of BMI classes and EOSS stages across different demographic subgroups. When assessing cause-specific mortality, deaths due to other causes were treated as competing events, and the results were reported as sub-distribution hazard ratios (SHRs) according to the model proposed by Fine and Gray [26].

A cut-off of 0.05 was adopted for the definition of statistical significance. Statistical analyses were performed using STATA 18 (StataCorp, College Station, TX, USA).

## 3. Results

### 3.1. Patient Characteristics

A total of 36,529 patients (18,424 males, 18,105 females) with a mean age of 47.7 ± 16.7 years were included in the analysis. Table 1 shows the main demographic and clinical characteristics of the patients at baseline, stratified according to BMI classes.

Subjects belonging to higher BMI categories were slightly younger, and with a higher prevalence of females. The percentage of non-Hispanic White and Hispanic individuals decreased with increasing BMI, while the percentage of non-Hispanic Blacks increased. Some differences were also observed in terms of income, with lower-income individuals being more represented in higher BMI categories. Notably, a significant correlation was found between BMI classes and EOSS stages; patients with no or subclinical weight-related medical conditions (EOSS stages 0–1) were more likely to belong to lower BMI classes, while the presence of established end-organ damage (EOSS stage 3) was more prevalent among patients with class III obesity.

### 3.2. All-Cause Mortality

After a median follow-up of 9.1 years (range: 0–20.8 years), 5329 deaths occurred; of these, 1431 were attributable to cardiovascular causes, 288 to cerebrovascular causes, 219 to diabetes-related complications, 135 to renal causes, 1243 to malignancies, and 2012 to other causes.

In the multivariable analysis (Table 2), both BMI classes and EOSS stages demonstrated an independent and complementary prognostic role in predicting all-cause mortality.

Specifically, a significantly increased mortality risk was observed both for class II and class III obesity (HR = 1.21, 95% CI 1.08–1.36, *p* = 0.001 and HR = 1.58, 95% CI 1.39–1.80, *p* < 0.001, compared to overweight, respectively), and for EOSS stage 2 and stage 3 (HR = 1.36, 95% CI 1.16–1.58, *p* < 0.001 and HR = 2.66, 95% CI 2.26–3.14, *p* < 0.001, compared to stage 0/1, respectively). When testing the role of demographic variables as effect modifiers for the relationship between BMI classes, EOSS stages, and overall mortality, a significant interaction was found between age and BMI (*p* = 0.002 for interaction), but not between age and EOSS (*p* = 0.364 for interaction). When the analysis was stratified by age groups, the relative hazard of death associated with higher BMI classes decreased progressively with age (Table 3).

Sex significantly influenced the association of both BMI (*p* = 0.001 for interaction) and EOSS (*p* = 0.046 for interaction) with all-cause mortality. In the stratified analyses, BMI classes showed a greater predictive role among male patients, while the discriminative ability of EOSS stages appeared to be greater in women (Table 4).

A borderline significance was found when testing the interaction between race/ethnicity and BMI (*p* = 0.063 for interaction), while no clear effect modification was found between race/ethnicity and EOSS (*p* = 0.135 for interaction); upon stratification, an apparent neutral effect of BMI categories on all-cause mortality was observed among non-Hispanic Black subjects in contrast to other ethnicities (Table 5).

### 3.3. Cause-Specific Mortality

Mortality due to cardiovascular causes was the leading cause of death in the study cohort and showed a similar pattern of association with BMI classes and EOSS stages as observed with all-cause mortality. In particular, an independent association was observed both for class II and class III obesity (SHR = 1.43, 95% CI 1.15–1.77, *p* = 0.001 and SHR = 1.97, 95% CI 1.52–2.56, *p* < 0.001, compared to overweight, respectively) and for EOSS stage 2 and stage 3 (SHR = 1.60, 95% CI: 1.09–2.35, *p* = 0.016 and SHR = 3.63, 95% CI 2.44–5.40, *p* < 0.001, compared to stage 0/1, respectively). The same held true also for mortality due to diabetes-related complications, which also demonstrated a statistically significant association with both class II–III obesity and EOSS stage 2–3. On the contrary, cerebrovascular mortality was not significantly predicted either by BMI classes or EOSS stages. EOSS stage 3 was the only predictor of death due to malignant neoplasms (SHR = 1.52, 95% CI 1.10–2.10, *p* = 0.025) or renal causes (SHR = 5.86, 95% CI 1.09–31.57, *p* = 0.040). The full results of the cause-specific mortality analyses are reported in Table 6.

## 4. Discussion

In this study, conducted on a representative sample of the general, non-institutionalized U.S. population, we observed an independent and complementary role of BMI and EOSS as predictors of all-cause and cause-specific mortality. Interestingly, a significant modulation of the effect was observed depending on the demographic characteristics of the patients studied: specifically, BMI demonstrated a greater predictive role in young patients, males, and individuals of non-Black ethnicity compared to their counterparts; on the other hand, the predictive capacity of the EOSS proved to be more stable across subgroups, although a gender difference was still noticeable, with a stronger prognostic role of EOSS in women compared to men.

### 4.1. All-Cause Mortality

The results of the present study suggest that including EOSS alongside BMI significantly enhances the prediction of all-cause mortality risk in PLwOW/O. These findings are consistent with those of a previous study, conducted on an earlier NHANES cohort (1988–1994), which also showed that individuals in EOSS stages 2 and 3 had an increased risk of all-cause mortality compared to those in stages 0/1 [21]. Similar findings were observed in the Aerobics Center Longitudinal Study, in which PLwO in EOSS stages 2 and 3 showed an increased risk of all-cause mortality compared to a control group composed of normal-weight individuals [20].

Clearly, the association between EOSS stages and mortality is not surprising, since this classification takes into account multiple conditions that are already known to be associated with mortality outcomes. However, it is interesting to note that, although the EOSS classification already summarizes most of the information regarding obesity-related comorbidities, BMI per se still retained an independent predictive role for the risk of all-cause mortality. In particular, consistent with the existing literature [11,12,13], significantly worse survival outcomes were observed for patients with obesity class II and III, independently of prevalent comorbidities classified by EOSS.

As already mentioned, concerns have been raised in recent years about the reliability of BMI in assessing the risk for obesity-related diseases [27,28,29]. With this in mind, EOSS was originally developed to provide a more comprehensive and individualized assessment of obesity-related health risks by focusing on the presence and severity of medical, mental, and functional complications associated with obesity. Nevertheless, the findings of this study highlight the fact that BMI and EOSS complement each other, each carrying a distinct and independent prognostic role in the prediction of overall mortality risk.

When examining the possible role of demographic variables as effect modifiers of the relationship between BMI, EOSS, and mortality, interesting insights emerged regarding how the predictive role of BMI and EOSS may vary by age, sex, and race/ethnicity. In fact, we observed a progressive decrease with age in the relative hazard of death associated with higher BMI classes. This result is consistent with the previous findings in the literature, where it was already observed that the association between BMI and mortality was attenuated as age increased [9,10], possibly related to the detrimental effects of malnutrition, sarcopenia or disease-induced unintentional weight loss in older adults [30,31,32], as well as to the so-called “survivor bias” [9]. In the present study, this heterogeneity between age groups was maintained when correcting the analyses for EOSS, thus isolating the effect of BMI alone, independent of obesity-related comorbidities.

When evaluating the possible heterogeneity of results according to race/ethnicity, a borderline-significant interaction between race/ethnicity and BMI was found. In the stratified analysis, this appeared to be mostly driven by individuals belonging to the non-Hispanic Black subgroup, in which BMI appeared to be essentially neutral with respect to all-cause mortality risk, in contrast to the findings in other ethnicities. The interpretation of this finding may probably lie in the well-known differences in the correlation between BMI and adiposity across race/ethnic groups [33]; at the same BMI value, in fact, Black individuals exhibit on average a higher free-fat mass [34], lower fat mass [35], and lower visceral adiposity [33]. Therefore, it is not fully surprising that BMI assessment may be less informative in this subgroup, being an even more inaccurate proxy for the definition of obesity and for the assessment of the risk of obesity-related complications.

With regard to sex, we observed that high BMI was more strongly associated with overall mortality in men than in women. Consistent with the findings of this study, other large previous epidemiologic studies had also already observed that the HRs linking BMI to mortality were higher in males than in females [9,10]. Similarly to age, in the present study we demonstrate that these gender differences persist also after adjusting the analyses for EOSS, thereby accounting for all obesity-related health conditions. This gender discrepancy may arise from physiological variations in body composition and fat distribution, as males exhibit greater abdominal adiposity and metabolic derangement compared to females at similar BMI values [36,37,38]. Interestingly, the analyses of this study also showed a gender difference in the predictive capacity of the EOSS, which showed a stronger prognostic role in women compared to men, thus possibly offsetting the lower stratification ability of BMI.

### 4.2. Cause-Specific Mortality

This study provided interesting insights into cause-specific mortality and its association with BMI classes and EOSS stages. Consistent with the existing literature, we found that cardiovascular mortality was the leading cause of death in this cohort. The observed independent association between higher BMI classes (specifically class II and III obesity) and increased cardiovascular mortality risk aligns with previous studies that have established a clear link between severe obesity and heightened cardiovascular risk [39]. Clinically, these findings underscore the importance of early and aggressive intervention in patients with class II–III obesity, particularly focusing on cardiovascular risk management. EOSS stages were also found to be strong predictors of cardiovascular mortality; of note, by its definition, EOSS summarizes the status of all major obesity-related cardiometabolic risk factors, thus pinpointing those patients with a higher global cardiometabolic burden. Nevertheless, the demonstration by the present study of a clear link between EOSS and cardiovascular mortality adds to the existing literature and further supports the use of EOSS as a useful guide for reliable risk stratification and targeted interventions.

In the context of diabetes-related complications, the findings of this study revealed a significant association with both class II–III obesity and EOSS stage 2–3. This is consistent with the established relationship between obesity and cardiometabolic comorbidities, with both higher BMI and concurrent metabolic alterations being known risk factors for the development and progression of diabetes-related complications [40,41].

Conversely, cerebrovascular mortality was not significantly predicted by either BMI classes or EOSS stages in this study. This finding diverges from previous evidence showing an increased risk of death due to stroke as BMI increases [9]. The results of the present study may be justified by the relatively low number of cerebrovascular deaths in our cohort (288 deaths), which may have resulted in insufficient statistical power for the assessment of this specific outcome.

Finally, EOSS stage 3 emerged as the sole predictor of mortality due to malignant neoplasms and renal causes. The association between EOSS stage 3 and deaths due to renal causes was not unexpected, as one of the components used for EOSS definition is the presence of chronic kidney disease. On the other hand, the association between obesity and cancer mortality goes into a debated topic with conflicting evidence. Obesity has been associated with increased mortality from any cancer [42,43], as well as from specific types of cancer, including prostate cancer in men [43,44] and breast cancer in women [45]. Conversely, better survival rates have been observed in PLwO diagnosed with melanoma, lung, and kidney cancer compared to patients with the same cancer without obesity [42]. The findings of this study showed a significant correlation between EOSS stage 3 and cancer mortality, while not observing a direct relationship with BMI classes, suggesting a possible link between cancer-related deaths and obesity-related comorbidities, rather than with BMI alone. However, given the aforementioned conflicting evidence in this regard and the impossibility of excluding with certainty the presence of residual confounds, larger studies, specifically having this as the primary endpoint, are advisable to confirm this finding and to better assess its possible clinical implications.

### 4.3. Strengths and Limitations

This study has several strengths. One is that it is a study conducted on a large, unselected, community-based sample extracted from the general U.S. population; its stratified, multistage, clustered probability design minimizes selection bias, thus enhancing the generalizability of the results. Another strength of this study is that, unlike most previous studies, we considered the possible role of demographic variables as effect modifiers of the relationship between BMI, EOSS, and mortality; the possibility to test for interactions and conduct stratified analyses provided interesting insights into how the predictive role of BMI and EOSS may vary by age, sex, and race/ethnicity. Finally, the large sample size of the cohort, as well as the long follow-up available, also allowed the assessment of cause-specific mortality, offering a more granular perspective on the prognostic role of BMI and EOSS classifications on the major obesity-related causes of death.

This study also has some limitations that must be acknowledged. First, although multivariable analyses accounted for all major factors that could possibly influence the risk of death, the possible presence of residual confounding cannot be completely ruled out. Second, the classification into EOSS classes had to be adapted to the available data, and the assessment of psychological functioning of the respondents could not be included. Third, some anamnestic data, although collected using structured questionnaires, relied on self-reporting, which may be subject to some degree of error and misclassification. Finally, the dietary patterns of participants, along with other important contributors to obesity, such as social influences and lifestyle habits, were not evaluated in this analysis.

## 5. Conclusions

The results of the present study observed an independent and complementary role of BMI and EOSS as predictors of all-cause and cause-specific mortality in PLwOW/O. Interestingly, the relationship between BMI, EOSS, and mortality was different depending on sex, age, and race/ethnicity as effect modifiers. Based on these data, the integrated use of these two classifications seems advisable to more comprehensively define the obesity-related risk of mortality across the full spectrum of the population.

## Figures and Tables

**Table 1:** Baseline characteristics of the subjects included in the analyses.

Variable	Patients with Overweight (N = 17,658)	Patients with Class I Obesity (N = 10,644)	Patients with Class II Obesity (N = 4769)	Patients with Class III Obesity (N = 3458)	*p*-Value
Age (years)	47.9 ± 17.1	48.3 ± 16.5	47.4 ± 16.1	45.4 ± 15.3	<0.001
Sex (%)					<0.001
Female	43.1	47.4	58.2	65.4	
Male	56.9	52.6	41.8	34.6	
Race/ethnicity (%)					<0.001
Non-Hispanic White	68.1	66.9	65.8	62.5	
Non-Hispanic Black	9.8	12.3	15.5	19.9	
Hispanic	15.7	15.9	14.9	12.8	
Other	6.4	4.9	4.8	4.8	
Annual household income (%)					<0.001
≥75,000 $	32.9	30.3	27.5	24.1	
45,000–74,999 $	21.1	21.8	21.5	21.4	
20,000–44,999 $	27.2	28.2	30.3	31.6	
<20,000 $	13.6	14.8	15.6	18.6	
Unknown	5.2	4.9	5.1	4.3	
Education level (%)					<0.001
More than high school graduate	56.7	54.8	54.3	55.0	
High school graduate	23.2	25.2	26.4	27.1	
Less than high school graduate	17.7	18.2	17.2	15.8	
Unknown	2.4	1.8	2.1	2.1	
Smoking status (%)					<0.001
Never smoker	52.1	52.8	53.5	56.8	
Former smoker	26.3	26.6	28.1	24.2	
Current smoker	19.9	19.4	17.1	17.8	
Unknown	1.7	1.2	1.3	1.2	
EOSS stage (%)					<0.001
Stage 0/1	34.7	25.1	21.4	18.8	
Stage 2	54.4	61.4	62.6	62.5	
Stage 3	10.9	13.5	16.0	18.7	

EOSS: Edmonton Obesity Staging System.

**Table 2:** Associations between all-cause mortality and BMI classes and EOSS stages.

	Overall Mortality (5348 Deaths)		
	HR	95% CI	*p*
BMI class			
Overweight	1 (reference)	-	-
Obesity Class I	1.06	0.98–1.16	0.139
Obesity Class II	1.21	1.08–1.36	0.001
Obesity Class III	1.58	1.39–1.80	<0.001
EOSS stage			
0/1	1 (reference)	-	-
2	1.38	1.16–1.58	<0.001
3	2.66	2.26–3.14	<0.001

Model adjusted for age, sex, ethnicity, income, education level, and smoking status. BMI: Body Mass Index; EOSS: Edmonton Obesity Staging System; HR: Hazard Ratio; CI: Confidence Interval.

**Table 3:** Subgroup analysis by age for all-cause mortality.

	<50 Years			50 ≤ Years < 70			≥70 Years		
	HR	95% CI	*p*	HR	95% CI	*p*	HR	95% CI	*p*
BMI class									
Overweight	1 (ref)	-	-	1 (ref)	-	-	1 (ref)	-	-
Obesity Class I	1.01	0.75–1.36	0.923	1.20	1.05–1.36	0.006	1.03	0.92–1.15	0.548
Obesity Class II	1.59	1.13–2.23	0.007	1.30	1.09–1.56	0.004	1.14	0.97–1.33	0.094
Obesity Class III	1.90	1.36–2.64	<0.001	1.75	1.40–2.20	<0.001	1.45	1.16–1.81	0.001
EOSS stage									
0/1	1 (ref)	-	-	1 (ref)	-	-	1 (ref)	-	-
2	1.54	1.15–2.05	0.003	1.40	1.04–1.89	0.024	1.24	0.95–1.64	0.110
3	3.07	2.10–4.49	<0.001	3.02	2.22–4.16	<0.001	2.28	1.72–3.02	<0.001

Models adjusted for age (as a continuous variable within age subgroups), sex, ethnicity, income, education level, and smoking status. BMI: Body Mass Index; EOSS: Edmonton Obesity Staging System; HR: Hazard Ratio; CI: Confidence Interval.

**Table 4:** Subgroup analysis by sex for all-cause mortality.

	Males			Females		
	HR	95% CI	*p*	HR	95% CI	*p*
BMI class						
Overweight	1 (ref)	-	-	1 (ref)	-	-
Obesity Class I	1.13	1.01–1.26	0.021	0.98	0.87–1.10	0.791
Obesity Class II	1.37	1.17–1.59	<0.001	1.04	0.90–1.22	0.537
Obesity Class III	2.03	1.62–2.53	<0.001	1.27	1.07–1.51	0.005
EOSS stage						
0/1	1 (ref)	-	-	1 (ref)	-	-
2	1.23	0.99–1.53	0.054	1.57	1.21–2.03	0.001
3	2.21	1.74–2.80	<0.001	3.45	2.60–4.58	<0.001

Models adjusted for age, ethnicity, income, education level, and smoking status. BMI: Body Mass Index; EOSS: Edmonton Obesity Staging System; HR: Hazard Ratio; CI: Confidence Interval.

**Table 5:** Subgroup analysis by race/ethnicity for all-cause mortality.

	Non-Hispanic White			Non-Hispanic Black			Hispanic			Other Ethnicity		
	HR	95% CI	*p*	HR	95% CI	*p*	HR	95% CI	*p*	HR	95% CI	*p*
BMI class												
Overweight	1 (ref)	-	-	1 (ref)	-	-	1 (ref)	-	-	1 (ref)	-	-
Obesity Class I	1.09	0.98–1.20	0.084	0.84	0.70–1.01	0.069	1.11	0.91–1.35	0.273	1.29	0.79–2.10	0.301
Obesity Class II	1.21	1.06–1.40	0.006	0.91	0.74–1.11	0.366	1.39	1.01–1.93	0.043	2.29	1.12–4.69	0.024
Obesity Class III	1.70	1.44–2.00	<0.001	1.11	0.91–1.36	0.278	1.45	1.05–1.99	0.021	1.81	0.70–4.68	0.220
EOSS stage												
0/1	1 (ref)	-	-	1 (ref)	-	-	1 (ref)	-	-	1 (ref)	-	-
2	1.30	1.06–1.60	0.011	2.07	1.55–2.76	<0.001	1.51	1.11–2.06	0.008	0.71	0.31–1.61	0.414
3	2.59	2.09–3.20	<0.001	4.13	2.92–5.84	<0.001	2.60	1.90–3.56	<0.001	1.06	0.46–2.41	0.893

Models adjusted for age, sex, income, education level, and smoking status. BMI: Body Mass Index; EOSS: Edmonton Obesity Staging System; HR: Hazard Ratio; CI: Confidence Interval.

**Table 6:** Associations between cause-specific mortality and BMI classes and EOSS stages.

	Cardiovascular Mortality (1431 Deaths)			Mortality Due to Malignancies (1243 Deaths)			Cerebrovascular Mortality (288 Deaths)			Mortality Due to DM-Related Complications (219 Deaths)			Mortality from Renal Causes (135 Deaths)			Mortality Due to Other Causes (1012 Deaths)		
	SHR	95% CI	*p*	SHR	95% CI	*p*	SHR	95% CI	*p*	SHR	95% CI	*p*	SHR	95% CI	*p*	SHR	95% CI	*p*
BMI class																		
Overweight	1 (ref)	-	-	1 (ref)	-	-	1 (ref)	-	-	1 (ref)	-	-	1 (ref)	-	-	1 (ref)	-	-
Obesity Class I	1.14	0.97–1.32	0.108	1.06	0.90–1.26	0.487	1.16	0.83–1.62	0.373	1.44	0.95–2.24	0.083	1.06	0.64–1.73	0.827	0.95	0.83–1.08	0.416
Obesity Class II	1.43	1.15–1.77	0.001	1.23	0.99–1.54	0.074	0.86	0.49–1.49	0.591	1.95	1.15–3.46	0.014	0.97	0.47–2.02	0.935	1.02	0.84–1.23	0.866
Obesity Class III	1.97	1.52–2.56	<0.001	0.97	0.70–1.33	0.837	0.92	0.50–1.69	0.800	4.29	2.68–7.80	<0.001	1.54	0.72–3.31	0.267	1.35	1.07–1.71	0.011
EOSS stage																		
0/1	1 (ref)	-	-	1 (ref)	-	-	1 (ref)	-	-	1 (ref)	-	-	1 (ref)	-	-	1 (ref)	-	-
2	1.60	1.09–2.35	0.015	1.30	0.97–1.73	0.081	1.05	0.44–2.48	0.914	11.93	2.77–51.35	0.001	3.35	0.66–16.91	0.143	1.46	1.12–1.89	0.004
3	3.63	2.44–5.40	<0.001	1.52	1.10–2.10	0.010	1.48	0.61–3.57	0.388	25.57	5.75–113.71	<0.001	5.86	1.09–31.57	0.040	2.21	1.67–2.91	<0.001

Models adjusted for age, sex, ethnicity, income, education level, and smoking status. BMI: Body Mass Index; EOSS: Edmonton Obesity Staging System; SHR: Sub-distribution Hazard Ratio; CI: Confidence Interval.

## Data Availability

The original data presented in the study are openly available in https://wwwn.cdc.gov/nchs/nhanes/Default.aspx.

## Supplementary Materials

The following supporting information can be downloaded at: https://www.mdpi.com/article/10.3390/nu16203433/s1, Table S1: Criteria for the classification of EOSS staging.
