# Supplementary material for: Complementary Role of BMI and EOSS in Predicting All-Cause and Cause-Specific Mortality in People with Overweight and Obesity
Source: Nutrients. 2024 Oct 10;16(20):3433. doi: 10.3390/nu16203433 (PMC11510653; doi:10.3390/nu16203433)
Supplement: Supplementary file 1 [file nutrients-16-03433-s001.zip › nutrients-3209762-supplementary.pdf]

Table S1. Criteria for the classification of EOSS staging.

Variable	EOSS 0	EOSS 1	EOSS 2	EOSS 3
Arterial hypertension	SBP < 130 mmHg **AND** DBP < 85 mmHg	SBP = 130-139.9 mmHg **OR** DBP = 85-89.9 mmHg	SBP ≥ 140 mmHg **OR** DBP ≥ 90 mmHg **OR** Self-reported diagnosis of hypertension **OR** Treatment with anti-hypertensive drugs	---
Diabetes mellitus	FG < 100 mg/dL **AND** HbA1c < 5.7%	FG = 100-125 mg/dL **OR** HbA1c = 5.7-6.4%	FG ≥ 126 mg/dL **OR** HbA1c ≥ 6.5% **OR** Self-reported diagnosis of diabetes **OR** Treatment with hypoglycemic drugs	---
Dyslipidemia	TC < 5.2 mmol/L **AND** HDL > 1.6 mmol/L **AND** TG < 1.7 mmol/L **AND** LDL < 3.4 mmol/L	TC = 5.2-6.1 mmol/L **OR** HDL = 1.0-1.6 mmol/L **OR** TG = 1.7-2.2 mmol/L **OR** LDL = 3.4-4.0 mmol/L	TC ≥ 6.2 mmol/L **OR** HDL < 1.0 mmol/L **OR** TG ≥ 2.3 mmol/L **OR** LDL ≥ 4.1 mmol/L **OR** Treatment with lipid-lowering drugs	---
Liver disease	No active liver disease and normal liver enzymes	No active liver disease but elevated liver enzymes	Active liver disease	---
Kidney disease	eGFR ≥ 90 ml/min/1.73m^2	eGFR = 60-89.9 ml/min/1.73m^2	eGFR = 30-59.9 ml/min/1.73m^2	eGFR < 30 ml/min/1.73m^2
Cardiovascular disease	---	---	---	Self-reported history of coronary heart disease, heart attack, angina pectoris, heart failure or stroke
Osteoarthritis	No history of joint or back pain	Occasional joint or back pain	Self-reported diagnosis of osteoarthritis	---
Physical health	No functional limitations	Mild functional limitations[a]	Moderate functional limitations[b]	Significant functional limitations[c]

DBP= diastolic blood pressure; eGFR= estimated glomerular filtration rate; FG=fasting glucose; HbA1c= glycated hemoglobin; HDL=high density lipoproteins; LDL=low density liporpoteins; SBP=systolic blood pressure; TC=total cholesterol; TG=triglycerides

[a] Patients were considered to have mild functional limitations if reporting some difficulty with walking for a quarter mile, walking up ten steps, stooping, crouching, kneeling, or lifting/carrying 10 pounds

[b] Patients were considered to have moderate functional limitations if reporting: (i) much difficulty with walking for a quarter mile, walking up ten steps, stooping, crouching, kneeling, or lifting/carrying 10 pounds; (ii) any difficulty with walking between rooms on the same floor, standing up from an armless chair, getting in and out of bed, using fork/knife/cup, or dressing themselves

[c] Patients were considered to have significant functional limitations if reporting inability to do any of the previously listed activities
